# Special issue on the 100^th^ anniversary of Xiamen University

**DOI:** 10.1038/s41377-021-00613-7

**Published:** 2021-09-14

**Authors:** Junyong Kang, Minghui Hong, Zhongqun Tian

**Affiliations:** 1 Engineering Research Center of Micro-nano Optoelectronic Materials and Devices, Ministry of Education, Fujian Key Laboratory of Semiconductor Materials and Applications, CI center for OSED, College of Physical Science and Technology, 361005 Xiamen, China; 2 Department of Electrical and Computer Engineering, National University of Singapore, 4 Engineering Drive 3, 117576 Singapore, Singapore; 3 State Key Laboratory of Physical Chemistry of Solid Surfaces, Collaborative Innovation Center of Chemistry for Energy Materials, College of Chemistry and Chemical Engineering, Xiamen University, 361005 Xiamen, China

**Keywords:** Optics and photonics, Lasers, LEDs and light sources

This special issue is devoted to the celebration of the century anniversary of Xiamen University (XMU) (6 April 2021) and the establishment of the LSA Editorial Office in Xiamen (3 July 2021), a collection to highlight the recent exciting research works performed in XMU or by XMU alumni, from all aspects of optics and photonics, including basic, applied and engineering research and applications. The guest editors are three XMU alumni who are active researchers in these areas: Professor Minghui Hong from National University of Singapore, Professor Zhongqun Tian and Professor Junyong Kang from XMU.

XMU is a renowned university continuously featuring creativity and dedication. It was the first university established by an overseas Chinese (Mr. Tan Kah Kee) and has been selected for the “Double First-class Initiative” Scheme, as one of the leading pioneers in achieving China’s ambition of building world-class universities. XMU is also the first prestigious Chinese university to set up an overseas branch campus (Xiamen University Malaysia), which has been honoured as the “shinning pearl” of “Belt and Road Initiative”. Rooted in XMU, the motto “Pursue Excellence, Strive for Perfection”, has been widely extended to all over the world through more than 400,000 of XMU graduates. During the century-long growth, XMU has achieved significant progress in the field of optics and photonics and become pioneers in several aspects: Development of laser holography that greatly promoted the anti-counterfeiting technology in China; study of entangled photon pairs that lead to quantum communications and optical micromanipulations, as well as the creation of facial recognition techniques based on the light beams; Development of surface-enhanced Raman spectroscopy (SERS) that not only conquers the material and surface limits of SERS, but also realizes the fast detection of trace hazards in the field of food safety; Improvement of efficiency for light-emitting diodes (LEDs) via creative pathways, including deep ultraviolet LEDs and high colour rendering warm-white LEDs. Research scholars across different disciplines in XMU are working on optoelectronic materials and devices, with interests in the designs and syntheses of luminescent materials, quantum dots, electrochromic materials as well as their applications in solid-state lighting, emissive displays, sensing, smart windows and photovoltaic cells. Experiments based on synchrotron-radiation light source as well as other advanced techniques are combined with computations/simulations to deeper understand the composition-structure-property coupling mechanism of functional materials, providing guidelines for developing high-performance materials and optoelectronic devices. This collection features some recent representative achievements in optical and optoelectronic sciences and technologies, contributed by XMU scholars and alumni. The innovative reports include: (1) Enhancement of deep ultraviolet emission by regulating the orbital state coupling^1^; (2) Development of mode-locked fibre lasers towards visible-wavelength^2^; (3) A new route for stress recording based on the Force-induced charge carrier storage effect in mechanoluminescence materials^3^; (4) Identification of unconventional host–guest complexation at nanostructured interface by surface-enhanced Raman spectroscopy^4^; (5) Quantification of electron accumulation at grain boundaries in perovskite polycrystalline films by correlative infrared-spectroscopic nanoimaging and Kelvin probe force microscopy^5^; (6) Improvement of detection in graphene/silicon heterojunction photodetectors through engineered tunnelling layer with enhanced impact ionization^6^; (7) Innovative approaches to determine the embedded electronic states at nanoscale interface via surface-sensitive photoemission spectroscopy^7^; (8) Development of AlGaN materials and optoelectronic devices that lead to the significant revolutions of solar-blind UV detection technology^8^; (9) Development of novel concepts and techniques in AlGaN-based LEDs for effectively controlling and tailoring the crucial properties of nitride quantum structures^9^; (10) Design of light-responsive and corrosion-resistant gas valve with non-thermal effective liquid-gating positional flow control^10^; (11) Design of nano/micro structures and macro-optical systems to enhance the detection sensitivity of surface-enhanced Raman and infrared spectroscopies^11^; (12) Demonstration of transmitting high-dimensional orbital angular momentum state at the single-photon level utilizing non-zero quantum discord^12^; (13) Development of hybrid laser processing technologies and their applications in the precision engineering of transparent hard materials^13^; (14) Demonstration of X-ray-charged bright persistent luminescence for multidimensional optical information storage by inkjet-printing NaYF_4_:Ln^3+^@NaYF_4_ nanoparticles^14^.

Enlightened by Light, Xiamen University, this 100-year-old university will become more elegant with greater academic excellence, especially in optics and photonics research.
